# Expression, purification, and characterization of galactose oxidase of *Fusarium sambucinum* in *E. coli*

**DOI:** 10.1016/j.pep.2014.12.010

**Published:** 2015-04

**Authors:** Regina Paukner, Petra Staudigl, Withu Choosri, Dietmar Haltrich, Christian Leitner

**Affiliations:** a Department of Food Science and Technology, BOKU-University of Natural Resources and Life Sciences, Vienna, Austria; b Department of Food Technology, Ramkhamhaeng University, Bangkok, Thailand

**Keywords:** Galactose oxidase, *Fusarium sambucinum*, Gene cloning, Heterologous expression, Steady-state kinetics, Alternative electron acceptors

## Abstract

A gene encoding a galactose oxidase (GalOx) was isolated from *Fusarium sambucinum* cultures and overexpressed in *Escherichia coli* yielding 4.4 mg enzyme per L of growth culture with a specific activity of 159 U mg^−1^. By adding a C-terminal His-tag the enzyme could be easily purified with a single affinity chromatography step with high recovery rate (90%). The enzyme showed a single band on SDS–PAGE with an apparent molecular mass of 68.5 kDa. The pH optimum for the oxidation of galactose was in the range of pH 6–7.5. Optimum temperature for the enzyme activity was 35 °C, with a half-life of 11.2 min, 5.3 min, and 2.7 min for incubation at 40 °C, 50 °C, and 60 °C, respectively. From all tested substrates, the highest relative activity was found for 1-methyl-β-galactopyranoside (226 U mg^−1^) and the highest catalytic efficiency (*k*_cat_/*K_m_*) for melibiose (2700 mM^−1^ s^−1^). The enzyme was highly specific for molecular oxygen as an electron acceptor, and showed no appreciable activity with a range of alternative acceptors investigated. Different chemicals were tested for their effect on GalOx activity. The activity was significantly reduced by EDTA, NaN_3_, and KCN.

## Highlights

• A galactose oxidase from *Fusarium sambucinum* was cloned and expressed in *E. coli*.
• It could be purified with a one step affinity chromatography step.
• Biochemical characteristics of the enzyme are comparable to galactose oxidases.

## Introduction

Galactose oxidase (GalOx^1^; d-galactose:oxygen 6-oxidoreductase, EC 1.1.3.9) is a monomeric 68-kDa enzyme that contains a single copper ion [1] and an amino acid-derived cofactor [2,3], formed by cross-linking of a Cys and a Tyr residue in the direct vicinity of the copper [4–6]. The thioether bond of the Tyr-Cys cross-link is post-translationally generated [4,7] and has been shown to affect the stability, the reduction potential [8] and the catalytic efficiency of the enzyme [9,10]. It has been classified as a member of the carbohydrate active-enzyme family AA5, subfamily 2 [11]. GalOx catalyzes the two-electron oxidation [3,12] of the C_6_-hydroxyl group of nonreducing d-galactose residues [13] as well as a range of primary alcohols to the corresponding aldehydes with concomitant reduction of oxygen to hydrogen peroxide [14–17]. During catalysis both the metal ion and the cysteine-modified tyrosine group undergo 1-electron redox interconversions [18]. Despite a wide substrate specificity, GalOx is strictly regioselective and no secondary alcohols are oxidized [19]. However, the enzyme accepts a wide variety of primary alcohols such as benzyl alcohol [20], and glycerol [21] as reducing substrates. GalOx displays remarkable stereospecificity in its reaction with sugars [22], being highly sensitive for the orientation of the C_4_-OH group, and hence it shows activity with galactose but not with glucose. Because of this specificity, various analytical techniques are based on GalOx, such as the determination of lactose in milk and dairy products [23] or the histochemical examination of mucus-secreting cells [24]. Furthermore, GalOx has been used in biosensors for the measurement of galactose and its derivatives in biological fluids [25], to label galactose residues in glycoconjugates [26], and for the induction of interferon in human lymphocyte culture [27,28]. GalOx is viewed as a competitive and cost-effective catalyst compared to chemical conversion for the manufacturing of fine chemicals for pharmaceutical purposes or in food industry, for example GalOx was used for conversion of sugars like d-galactose to food-grade cross-linking agents [29–32]. Another important application for GalOx is the modification of cell surface carbohydrates and has been used in cell labeling studies and histochemical staining [19]. GalOx is interesting for the use in industrial processes such as derivatization of guar gum and related polymers as well [33,34].

The enzyme is secreted by a number of fungal species, of which *Fusarium graminearum* (formerly classified as *Dactylium dendroides*) is the most extensively studied [35–43]. The production and purification of GalOx has been reported from its natural fungal source [26,39,44–49], furthermore, various GalOx genes were cloned and successfully expressed in the filamentous fungi *Aspergillus nidulans* [50,51], *Aspergillus* *oryzae* and *Fusarium venenatum* [52], which have no endogenous GalOx, in the methylotrophic yeast *Pichia pastoris* [4,10,33,36,53–56] and in the bacterium *Escherichia coli* [55,57–59]. Typically, wild-type fungal GalOx is produced as a preproform carrying an N-terminal signal sequence, which is removed upon secretion, yielding the immature proform. The prosequence in this form was suggested to function as an intramolecular chaperone supporting copper binding and cofactor formation [42,50]. The maturation of GalOx requires several successive steps including cleavage of the signal sequence, which directs translocation, metal binding and cofactor processing [12,43]. Subsequently, the prosequence is removed and the Tyr-Cys cofactor is formed by self-processing reactions [2,7].

In the present paper we describe cloning and recombinant expression of a new *gao* gene without its prepro sequence from *Fusarium sambucinum* in *E. coli*. Furthermore, the purification and biochemical characterization of the enzyme are reported. Alternative electron acceptors, and possible activators as well as inhibitors were tested for their effect on GalOx activity.

## Materials and methods

### Chemicals, strains and vectors

Chemicals for enzyme assays, buffers and media were purchased from Sigma–Aldrich (Steinheim, Germany) and were of the highest purity available. 2,2′-Azino-bis(3-ethylbenzthiazoline-6-sulfonic acid) (ABTS) was purchased from Amresco (Solon, OH, USA). Restriction enzymes, dNTP mix, Rapid DNA Ligation Kit and standard for Agarose gelelectrophoresis (GeneRuler DNA Ladder Mix) were from Fermentas (Vilnius, Lithuania) and the Phusion polymerase was from New England BioLabs (Ipswich, UK). Synthetic oligonucleotides were synthesized by VBC-Biotech (Vienna, Austria). *E.* *coli* strain BL21 (DE3) was purchased from Invitrogen (Carlsbad, CA, USA), the cloning vector pJET 1.2 was from Fermentas and the expression vector pET21a was from Novagen (Madison, WI, USA). The HisPrep FF 16/10 column was from GE Healthcare Bioscience AB (Uppsala, Sweden). SDS–PAGE protein standard (Precision Plus Protein prestained standard) was from BioRad (Herts, UK). The electron acceptors ferrocenium (FcPF_6_), guaiacol, 2,6-dimethoxyphenol, caffeic acid, p-coumaric acid, ferulic acid, sinapic acid, Thioflavin T, 2-(4′-methylaminophenyl)benzothiazole, 1,1′-diethyl-2,2′-carbocyanine iodide, 1,4-benzoquinone, 2,6-dichloro-indophenol, and ferricyanide were purchased from Sigma–Aldrich. *F.* *sambucinum* (synonym *Gibberella pulicaris*) strain MA1886 was kindly provided by Gerhard Adam (Department of Applied Genetics and Cell Biology, BOKU Vienna, Austria).

### Isolation and cloning of the GalOx gene

*F. sambucinum* MA1886 was cultivated in 50 mL Sabouraud medium (5 g L^−1^ peptone from casein, 5 g L^−1^ peptone from meat, 10 g L^−1^ glucose, 10 g L^−1^ maltose, 5 g L^−1^ yeast extract) in shaken flasks at 25 °C and 110 rpm for 3 days. Fungal mycelia were collected by centrifugation at 4 °C and 5000×*g* for 15 min and the pellet was washed in 50 mL saline solution (5 g L^−1^ NaCl, 0.12 g L^−1^ MgSO_4_·7H_2_O). Genomic DNA was isolated from 100 mg of frozen mycelia ground in liquid nitrogen by the phenol–chloroform-extraction as described by Chomczynski and Sacchi [60]. The *gao* gene coding for GalOx was amplified by PCR using degenerated primers based on the published sequences from related organisms (Accession Number: FGSG_11032.3/M86819/FOXG_09956.2/FVEG_08555.3): 5′-GCCTCAGCA/TCCC/TA/CTCGG-3′ and 5′-CTGAGTAACGA/CGAAG/TA/CGT-3′, purified by agarose gel electrophoreses and subcloned into the pJET 1.2 cloning vector using the CloneJET PCR Cloning Kit (Fermentas). Restriction sites were introduced using the following forward primers: 5′-TCGCACATATGTACCTTTTGTCACTCGCTC-3′ and 5′-GCTGACATATGGCCTCAGCACCCATTGGA-3′ for *gao* with and without the prepro sequence, respectively, and 5′-GCTACGCGGCCGCCTGAGTAACGCGAAT-3′ as the reverse primer (restriction sites underlined). Subsequent, the PCR product was digested with *Nde*I and *Not*I and cloned in the equally treated expression vector pET21a in frame with the C-terminal His_6_-tag by the Rapid DNA Ligation Kit. The resulting plasmid was transformed into *E. coli* BL21 (DE3) by electroporation. DNA sequencing was performed as a commercial service (LGC Genomics; Berlin, Germany). The amino acid sequence derived from the GalOx gene was used to generate a three-dimensional model based on the published structure of GalOx from *F. graminearum* [5] using SWISS-MODEL [61–63].

### Heterologous expression and purification

Cultivation of *E. coli* BL21 (DE3) for production of the recombinant enzyme was performed in 30 mL of double concentrated LB medium (20 g L^−1^ peptone from casein, 10 g L^−1^ yeast extract and 10 g L^−1^ NaCl) with 50 mg L^−1^ ampicillin in 125-mL baffled flasks. Cells were grown at 37 °C and 120 rpm until reaching an OD_600_ of 0.4–0.6. Then recombinant protein expression was induced by addition of 5% lactose and cultivation was continued at 25 °C and 130 rpm overnight. The cell pellet after centrifugation was resuspended in 20 mM potassium phosphate buffer pH 7.0, and an aliquot of 500 μL was homogenized by Precellys24 (PEQLAB, Erlangen, Germany). The cell homogenate was tested for the presence of GalOx activity. Large scale cultivation was done in 1-L baffled flasks containing 300 mL medium [59].

The biomass from these cultivations was harvested by centrifugation at 4000×*g* for 20 min and 4 °C, and resuspended in phosphate buffer (20 mM, pH7.0). After disruption in a French Press at 100 MPa the crude cell extract was separated from cell debris by centrifugation (30,000×*g*, 4 °C, 30 min) and used for protein purification by Immobilized Metal Affinity Chromatography (IMAC) with a 20 mL Ni-charged Sepharose 6 Fast Flow column (HisPrep FF 16/10; GE Healthcare). Before loading the sample the column was equilibrated with 10 column volumes (CV) of buffer A (20 mM KH_2_PO_4_, 1 M NaCl, 10 mM imidazole, pH 8.0). After the protein sample was applied to the column, it was washed with 3 CV of the same buffer, and eluted in a linear gradient from 0.01 to 1 M imidazole in 10 CV. Fractions containing GalOx activity were pooled and the purity of the purified GalOx was checked by electrophoresis. SDS–PAGE was performed in principle as described by Laemmli [64] using the PerfectBlue vertical electrophoresis apparatus (PEQLAB) and the Precision Plus Protein Dual Color kit as mass standard. Proteins were visualized by Coomassie brilliant blue staining.

### Enzyme activity assay

Prior to activity measurement GalOx was activated by incubation with 1 mM CuSO_4_ for 30 min at 800 rpm and 25 °C. GalOx was measured with the chromogenic ABTS (2,2′-azinobis(3-ethylbenzthiazolinesulfonic acid)) assay [65]. The absorbance change at 420 nm (*ε*_420_ = 43.2 mM^−1^ cm^−1^) was recorded at 30 °C for 180 s. The standard assay mixture (total volume, 1 mL) contained 1 μmol of ABTS in 20 mM potassium phosphate buffer (pH 7.0), 2 U horseradish peroxidase, 100 μmol d-galactose, and a suitable amount of GalOx sample. One Unit of GalOx activity was defined as the amount of enzyme that is necessary for the oxidation of 2 μmol of ABTS per min, which equals the consumption of 1 μmol of O_2_ per min, under the conditions described above. Protein concentrations were determined at 595 nm by the Bradford assay [66] using the BioRad Protein Assay Kit with BSA as standard.

### pH dependence of activity

A pH–activity profile was determined in the range of pH 2.5–10.0 using the buffer systems citric acid (pH 2.5–6.0), potassium phosphate (pH 6.0–8.0) and Tris (pH 8.0–10.0), each at a concentration of 50 mM. Activity measurements were performed otherwise as described for the standard assay.

### Temperature optimum and thermal stability

Determination of the temperature optimum of GalOx was achieved by measuring the activity with the standard assay at different temperatures in the range of 30–70 °C. Thermal stability of GalOx was determined by incubating the protein at 30, 40, 50 and 60 °C. Samples were taken at various time points, cooled on ice, and residual GalOx activity was measured using the standard ABTS assay after reactivating the enzyme by incubation with CuSO_4_.

### Steady-state kinetic measurements

Steady-state kinetic constants were measured for GalOx for different electron donor substrates. All kinetic measurements were performed at 30 °C in 20 mM phosphate buffer (pH 7.0). Measurement of kinetic constants for various sugar substrates were done with oxygen (air saturation) and the standard ABTS assay. d-Galactose (1–500 mM), 1-methyl-β-galactopyranoside (5–200 mM), melibiose (1–250 mM), raffinose (10–250 mM), and lactose (5–250 mM) were used as substrate. Kinetic constants were calculated by nonlinear least-square regression, fitting the data to the Henri–Michaelis–Menten equation (Sigma Plot 9, Systat; Chicago, IL, USA).

### Alternative electron acceptors

The enzyme reactions were carried out in a glove box (Whitley DG250, Don Whitley Scientific, Shipley, UK) and were followed spectrophotometrically using an Agilent 8453 UV–visible spectrophotometer (Agilent Technologies, Santa Clara, CA, USA) at 30 °C, and quantified at wavelengths indicated later in the manuscript (Table 3). To eliminate oxygen the glove box was evacuated and flushed with a nitrogen/hydrogen mixture (99/1) repeatedly. Residual oxygen was removed with the built-in palladium catalyst and the produced water vapor absorbed with silica gel. All buffers and reagents used were flushed with the same gas mixture.

As possible alternative electron acceptors for GalOx, the following compounds were tested: ferrocenium ion (FcPF_6_), 1,4-benzoquinone, 2,6-dichloro-indophenol (DCIP), and ferricyanide. The activity test was performed with 0.2 U GalOx as with the standard assay, but using the respective electron acceptor instead of oxygen. The reaction stoichiometry is one for the two-electron acceptors (1,4-benzoquinone and DCIP) and two for the one-electron acceptors (ferrocenium ion and ferricyanide). Furthermore, the oxidized, radical forms of the ABTS cation, the phenols guaiacol, 2,6-dimethoxyphenol (DMP), caffeic acid, p-coumaric acid, ferulic acid and sinapic acid, the benzothiazoles thioflavin T and 2-(4′-methylaminophenyl)benzothiazole (BTA-1) and the cyanine dye 1,1′-diethyl-2,2′-carbocyanine iodide were tested as electron acceptors. Due to the short lifetime of these radicals they were produced immediately before the analyses. The oxidation of the corresponding compound to the radical was performed by recombinant laccase from *Botrytis aclada* expressed in *P.* *pastoris* [67] in stirred quartz cuvettes containing 1 mM of the radical builder, 100 mM d-galactose and 1.5 U laccase in 20 mM potassium phosphate buffer pH 6.5 in a final volume of 2.7 mL. The reaction was followed spectrophotometrically. After the oxidation was completed, laccase was inhibited by adding 300 μL 10 mM NaF solution, oxygen was removed by flushing with nitrogen, and the reaction was started immediately by adding 0.2 U GalOx to 1 mL of the radical solution. The negative control was performed without GalOx. For a positive control ascorbate (1.25 mM) was added instead of GalOx.

### Effect of various compounds on GalOx activity

The effect of various compounds on the activity of purified GalOx was evaluated by performing the enzyme assay with the addition of 5 mM of each substance (Table 4) except Tween80, which was used in a concentration of 2.5%. Before measuring the activity the enzyme sample was incubated in the assay buffer containing the tested substance and ABTS for 5 min at 30 °C and the reaction was then started by addition of d-galactose.

## Results and discussion

### Isolation and heterologous expression of GalOx-encoding gene

**Fig. 1:**
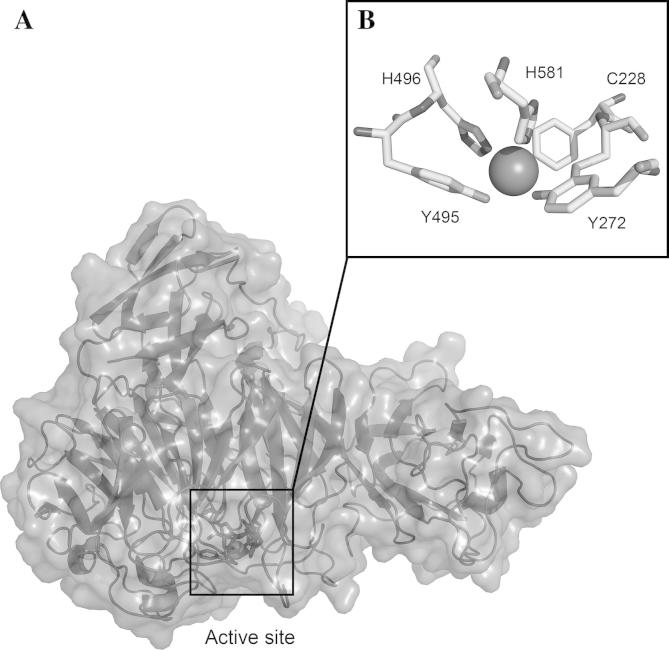
3D structure of GalOx of *F. sambucinum*. (A) Overall structure showing the predominantly β-structure. (B) The active site of GalOx showing the copper ligands and the thioether cross-link. The structural model was generated by homology modeling based on the published structure of mature GalOx from *F. graminearum* (PDB 1gog) using SWISS_MODEL.

Mycelium of *F. sambucinum* from a culture grown in liquid medium was harvested and the genomic DNA was isolated. Degenerated primers based on published sequences were used to amplify the *gao* gene coding for GalOx including its signal sequence. The gene consists of an open reading frame of 2037 bp encoding a polypeptide of 679 amino acids. The sequence (GenBank accession No. KM052576) contains no introns and a 37 amino acid prepro sequence. The similarity to the protein sequences of GalOx from *F. graminearum* [5] and *Fusarium oxysporum*[68] are 96% and 81%, respectively. The amino acid sequence derived from the *F. sambucinum gao* gene was used to generate a three-dimensional homology model based on the published structure of mature GalOx (1gog) from *F. graminearum* [5] using SWISS-MODEL [61–63] (Fig. 1). The amino acids in the active site (Fig. 1B) as well as in the second shell surrounding it are completely conserved. The architecture of the substrate-binding pocket as well as the residues responsible for copper binding are also identical. The changes in amino acid sequences are mainly found on the surface of the protein.

Based on the determined nucleotide sequence, modified oligonucleotide primers containing restriction sites for *Nde*I and *Not*I were constructed. The gene was cloned with and without its prepro sequence into the expression vector pET21a, adding a C-terminal His_6_ tag to the protein. After transformation of the plasmids into *E. coli* BL21(DE3) different clones were selected, cultivated on a small scale in double-concentrated LB medium, and 5% of lactose was used as inducer for expression of the *gao* gene with and without its prepro sequence. No active enzyme was found in the clones containing the full-length *gao* gene containing its prepro sequence. From the clones containing the *gao* gene without its prepro sequence the best producer was selected and used for larger scale production of GalOx in 1-L shaking flasks. Routinely, 4.4 mg L^−1^ of active, soluble GalOx (as calculated from a volumetric activity of 704 U L^−1^ and a specific activity of the homogenous enzyme of 159 U mg^−1^) were obtained in shaking flask cultivation after incubation at 25 °C for 16 h. This translates to a space–time yield of 0.28 mg h^−1^ L^−1^.

### Enzyme purification and molecular properties

**Fig. 2:**
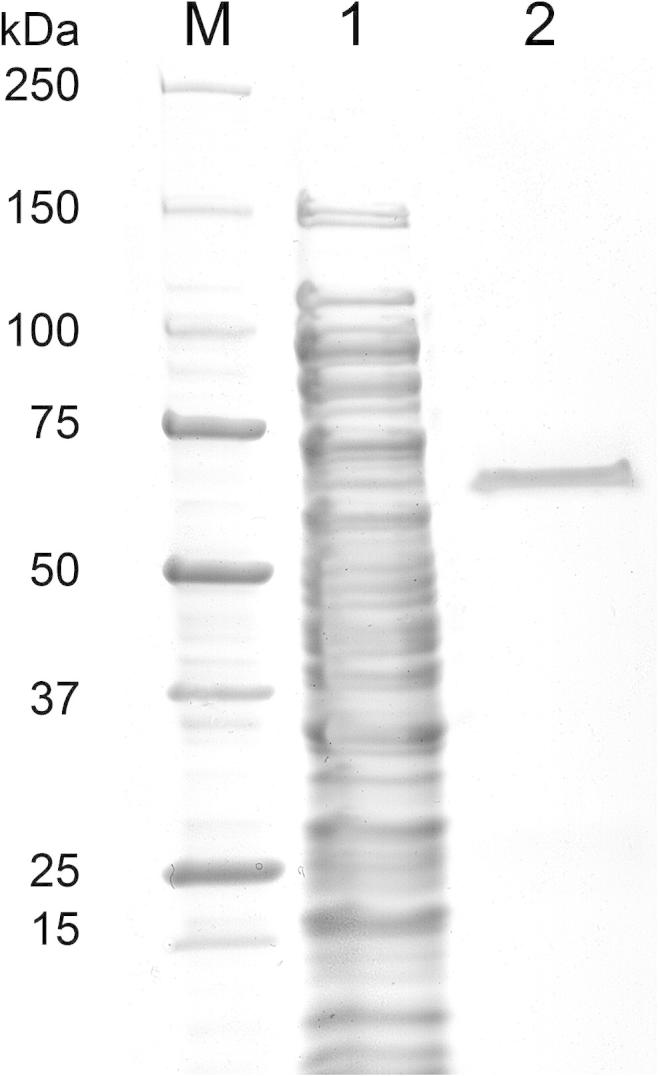
SDS–PAGE analysis of GalOx from *F. sambucinum* produced in *E. coli*. Lane M, precision plus protein standard (BioRad); lane 1, crude cell extract; lane 2, purified GalOx after IMAC.

Recombinant GalOx was purified 204-fold from the crude cell extract in one single chromatographic step by Immobilized Metal Affinity Chromatography using a HisPrep column as outlined in Table 1. Even strict pooling of only the purest fractions resulted in a high yield of 91% and the final recombinant GalOx preparation had a specific activity of 159 U mg^−1^. The purification procedure yielded an enzyme preparation that was apparently homogenous as judged by SDS–PAGE (Fig. 2), which shows a single band. As estimated from this SDS PAGE, GalOx has an apparent molecular mass of 68.5 kDa. When compared to the calculated molecular mass of 70.3 kDa derived from the amino acid sequence, SDS–PAGE underestimates the molecular mass by 2.5%. The faster migration indicates that the thioether bond between Cys228 and Tyr272 is formed in the enzyme [7,51].

### Kinetic properties

**Fig. 3:**
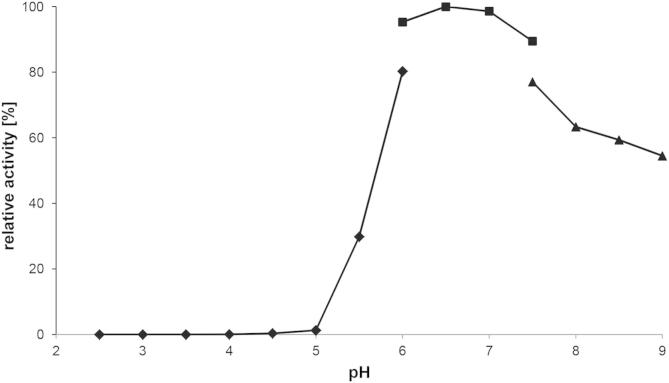
Effect of the pH on the activity of GalOx from *F. sambucinum* produced in *E. coli*. The buffers used were 50 mM citrate (●), 50 mM phosphate (■) and 50 mM Tris (▴).

The pH and temperature optima were determined using d-galactose as electron donor and oxygen as electron acceptor. The pH-profile (Fig. 3) for recombinant GalOx is rather broad with a bell-shaped curve, showing more than 95% activity in the range of pH 6–7.5. Below pH 5 GalOx shows no activity. This is in good agreement with data reported previously for native GalOx from different sources [44,48,49].

**Fig. 4:**
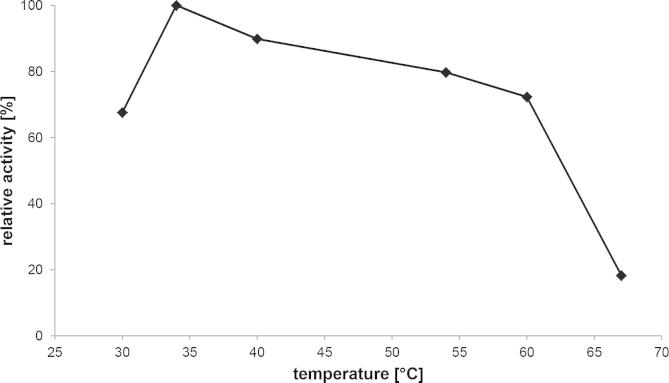
Temperature optimum of GalOx from *F. sambucinum* produced in *E. coli*.

**Fig. 5:**
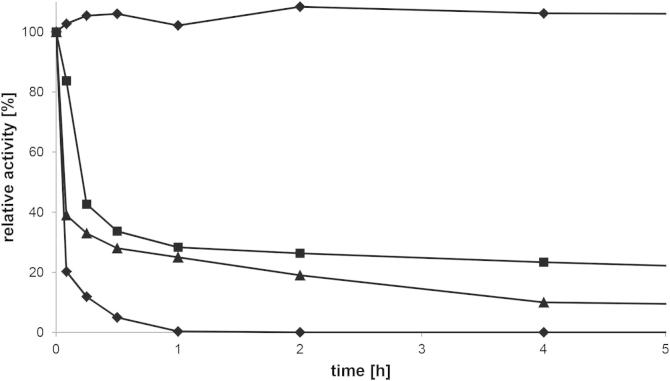
Thermal stability of GalOx from *F. sambucinum* produced in *E. coli*. Pre-incubation of GalOx at 30 °C (●), 40 °C (■), 50 °C (▴) and 60 °C (♦), respectively, for various time points.

A temperature optimum of 35 °C for purified GalOx was estimated by measuring the activity at various temperatures (Fig. 4). The enzyme was active up to 70 °C during the 3-min assay. The thermal stability was determined by incubating purified GalOx at various temperatures (30 °C, 40 °C, 50 °C, and 60 °C) and measuring the residual activity of aliquots taken at the times indicated. The results (Fig. 5) show that the enzyme is stable at 30 °C for at least 24 h of incubation. The calculated half-life was 11.2 min, 5.3 min, and 2.7 min for incubation at 40 °C, 50 °C, and 60 °C, respectively. The thermostability of GalOx from *F. sambucinum* is therefore significantly lower than reported for GalOx from other *Fusarium* strains [49,57].

GalOx has a broad substrate specificity, which is one of the most interesting characteristics of the enzyme [19]. Steady-state kinetic constants for various substrates were determined using oxygen (air) as electron acceptor. The initial rates of substrate turnover were recorded using different substrate concentrations in the standard ABTS assay at 30 °C and pH 7.0. Kinetic data are summarized in Table 2. The enzyme showed the highest relative activity with 1-methyl-β-galactopyranoside (142% relative to galactose) and approx. 80% relative activity with melibiose, raffinose, and lactose. The highest catalytic efficiency (*k*_cat_/*K_m_*) was found for melibiose (2700 M^−1^ s^−1^) followed by raffinose (2500 M^−1^ s^−1^) whereas the corresponding value for d-galactose was 3-fold lower. The lowest catalytic efficiency was measured for lactose as a result of an unfavorably high Michaelis constant of 683 mM. The lowest measured *K_m_* value, 16 mM for melibiose, is still rather high when compared to other carbohydrate-active enzymes, which seems to be due to the broad substrate specificity for GalOx [33].

It is of interest to know whether GalOx can transfer electrons to other acceptors than oxygen. To answer this question we tested a range of alternative electron acceptors, some of which are used by other copper-containing oxidoreductases. The one-electron acceptors included also different organic radicals. Due to the short life span of these radicals they were produced directly prior to their use by oxidation with laccase, which was inhibited with fluoride when the radical-forming reaction was completed. GalOx did not show significant activity with any of the tested electron acceptors (Table 3). These results are similar to those published by Aisaka et al., who tested different possible electron acceptors but also failed to find an alternative to oxygen [69]. GalOx is therefore a true oxidase without detectable dehydrogenase activity.

The effect of various compounds, mainly various metal ions, on GalOx activity was determined (Table 4). Monovalent and divalent cations like Mg^2+^, K^+^, Na^+^, NH_4_^+^, and Mn^2+^ showed no significant effect on the enzyme activity. The nonionic detergent Tween80 and fluoride had also no effect on GalOx activity. The enzyme activity was reduced to less than 50% by EDTA. This result is different to published data [44,46,49] for GalOx from *Fusarium acuminatum*, *Gibberella fujikuroi* and *Polyporus circinatus*, respectively, where EDTA did not inhibit activity significantly. As expected other metallo-enzyme inhibitor such as azide and cyanide completely inactivated GalOx.

## Conclusions

GalOx is of interest for a number of biotechnological applications. Because of this interest, more detailed knowledge about GalOx from different sources is important since this might reveal novel or improved areas of application. The *gao* gene coding for GalOx from *F. sambucinum* can be easily expressed in the bacterial expression host *E. coli* even without codon optimization. A simple one step affinity purification is sufficient to purify the protein with a yield of 90%. GalOx from *F. sambucinum* is very well comparable in its biochemical and catalytic properties to other fungal GalOx, which is not surprising when considering the well-conserved geometry of the active site and the substrate-binding site in these enzymes. Because of its similar biochemical properties and its simple and efficient purification protocol this new enzyme could be an alternative for GalOx from other sources.

## Conflict of interest

The authors declare no conflict of interest.

## Figures and Tables

**Table 1:** Purification of recombinant GalOx from *F. sambucinum* by immobilized metal affinity chromatography (IMAC).

Purification step	Total protein (mg)	Total activity (U)	Specific activity (U/mg)	Purification (fold)	Yield (%)
Crude-extract	210	165	0.78	1	100
IMAC	0.95	150	159	204	91

**Table 2:** Apparent kinetic constants of GalOx from *F. sambucinum* produced in *E. coli* for several electron donors.

Substrate	*V*_max_ (μmol min^−1^ mg^−1^)	*K_m_* (mM)	*k*_cat_ (s^−1^)	*k*_cat_/*K_m_* (M^−1^ s^−1^)
d-Galactose	47 ± 1.1	61 ± 4.2	54	890
1-Methyl-β-galactopyranoside	67 ± 1.0	39 ± 1.7	77	2000
Lactose	38 ± 4.2	683 ± 95.5	44	64
Melibiose	37 ± 0.8	16 ± 1.5	42	2700
Raffinose	43 ± 1.2	20 ± 2.3	49	2500

**Table 3:** Effect of various alternative electron acceptors on the activity of GalOx.

Electron acceptor	Conc. stock (mM)	*A*_max_ (nm)	Absorption coefficient (mM^−1^ cm^−1^)	Relative activity (%)
O_2_	Air	420	43.2	100
ABTS cation radical	1	420	43.2	0
Ferrocenium ion	1	300	4.3	0
1,4-Benzoquinone	10	290	2.24	0
2,6-Dichloro-indophenol (DCIP)	3	600	11.8	0
Ferricyanide	4	420	0.98	0
Guaiacol radical	10	465	12.1	0
2,6-Dimethoxyphenol radical	10	469	49.6	0
Caffeic acid radical	10	315	n.d.	0
p-Coumaric acid radical	10	285	n.d.	0
Ferulic acid radical	10	285	n.d.	0
Sinapic acid radical	10	305	n.d.	0
Thioflavin T	10	220, 400, 490, 950	n.d.	0
2-(4′-Methylaminophenyl)benzothiazole (BTA-1)	10	350	n.d.	0
1,1′-Diethyl-2,2′-carbocyanine iodide	10	420, 614	n.d.	0

**Table 4:** Effect of various compounds on GalOx activity. GalOx was incubated 5 min at 30 °C with the compound before measuring activity.

	Relative activity (%)
MgCl_2_	104
KCl	109
NaCl	98
NH_4_Cl	98
MnCl_2_	96
NaF	103
KCN	<1
NaN_3_	<1
EDTA	43
Tween80	110

## Appendix A Supplementary data

Supplementary data GalOx_*F*_*sambucinum*_model.

## Footnotes

1 *Abbreviations used:* GalOx, galactose oxidase; IMAC, immobilized metal affinity chromatography; SDS–PAGE, one dimensional sodium dodecylsulfate polyacrylamide gel electrophoresis; ABTS, 2,2′-azinobis (3-ethylbenzthiazolinesulfonic acid); DCIP, 2,6-dichloro-indophenol; FcPF_6_, ferrocenium; DMP, 2,6-dimethoxyphenol; BTA-1, 2-(4′-methylaminophenyl)benzothiazole.
